# Sustainable biosurfactant production from secondary feedstock—recent advances, process optimization and perspectives

**DOI:** 10.3389/fchem.2024.1327113

**Published:** 2024-01-19

**Authors:** Yahui Miao, Ming Ho To, Muhammad Ahmar Siddiqui, Huaimin Wang, Sofie Lodens, Shauhrat S. Chopra, Guneet Kaur, Sophie L. K. W. Roelants, Carol Sze Ki Lin

**Affiliations:** ^1^ School of Energy and Environment, City University of Hong Kong, Kowloon, China; ^2^ Branch of Chinese National Engineering Research Center for Control and Treatment of Heavy Metal Pollution, The Hong Kong University of Science and Technology, Kowloon, China; ^3^ McKetta Department of Chemical Engineering, Cockrell School of Engineering, The University of Texas at Austin, Austin, United States; ^4^ Bio Base Europe Pilot Plant, Ghent, Belgium; ^5^ Centre for Industrial Biotechnology and Biocatalysis (InBio.be), Faculty of Bioscience Engineering, Ghent University, Ghent, Belgium; ^6^ School of Engineering, University of Guelph, Guelph, ON, Canada

**Keywords:** glycolipid, valorization, biorefinery, biosurfactant, sustainability, secondary feedstock

## Abstract

Biosurfactants have garnered increased attention lately due to their superiority of their properties over fossil-derived counterparts. While the cost of production remains a significant hurdle to surpass synthetic surfactants, biosurfactants have been anticipated to gain a larger market share in the coming decades. Among these, glycolipids, a type of low-molecular-weight biosurfactant, stand out for their efficacy in reducing surface and interfacial tension, which made them highly sought-after for various surfactant-related applications. Glycolipids are composed of hydrophilic carbohydrate moieties linked to hydrophobic fatty acid chains through ester bonds that mainly include rhamnolipids, trehalose lipids, sophorolipids, and mannosylerythritol lipids. This review highlights the current landscape of glycolipids and covers specific glycolipid productivity and the diverse range of products found in the global market. Applications such as bioremediation, food processing, petroleum refining, biomedical uses, and increasing agriculture output have been discussed. Additionally, the latest advancements in production cost reduction for glycolipid and the challenges of utilizing second-generation feedstocks for sustainable production are also thoroughly examined. Overall, this review proposes a balance between environmental advantages, economic viability, and societal benefits through the optimized integration of secondary feedstocks in biosurfactant production.

## 1 Introduction

Surfactants, consisting of a hydrophobic tail and a hydrophilic head, play a crucial role in various applications (Ahmar Siddiqui et al., 2022). These amphiphilic compounds aggregate at liquid-oil interfaces, reducing the surface tension. Their unique properties, including dispersion, emulsification, and biological activities (Ines and Dhouha, 2015; Madankar and Meshram, 2022), have led to their extensive use in detergents, foam-forming agents, emulsifiers, and wetting agents and the formulation of diverse products such as household cleaners, industrial solutions, food additives, healthcare products, paints, oil remediation agents, and printing materials (Johnson et al., 2021). Surfactants have also found application in petroleum and penetrant industries, improving water and treatment agent distribution in underground rocks for enhanced oil recovery rates (ORR) (Santos et al., 2016). Extensive research and development have explored surfactant applications across multiple fields, including pharmaceutics, cosmetics, nanotechnology, optoelectronics, bioremediation, chemical transformation, and drug delivery (Sachdev and Cameotra, 2013; Karlapudi et al., 2018). Based on their ionic properties and behaviour in aqueous solutions, surfactants can be further classified into anionic, cationic, amphoteric and zwitterionic surfactants (Os van et al., 2012). This classification aids in understanding and predicting their performance in various applications such as detergency, fabric care, emulsification, germicide, foaming, and solubilization (De Guertechin, 1999). Anionic surfactants, in particular, exhibit effective interaction with positively charged water pollutants, leading to their dispersion and dissolution (Cheng et al., 2020), making them the preferred choice for industrial and household cleaning, accounting for a significant 50% share (Barra Caracciolo et al., 2017). In addition, anionic surfactants are also popular in cosmetic products due to their superior properties, including foaming, cleansing, thickening, solubilising, emulsifying and antimicrobial effects (Mordor Intelligence, 2022).

It has been estimated that the surfactant market will surpass USD 58.3 billion by 2024 and USD 81.7 billion by 2030, with an annual growth rate of 4.5%–5% (Precedence Research, 2022). This growth can be attributed to several factors, including the global population growth and the impact of the COVID-19 pandemic (Chirani et al., 2021). While the pandemic has negatively affected the market, with reduced demand for fuel additives and associated surfactants due to the lockdown measures (Siddique et al., 2021), it has also increased public awareness of personal hygiene and cleanliness. This heightened consciousness has stimulated the demand for personal and household cleaning products, contributing to the overall growth of the cleaning-related surfactant industry (Alygizakis et al., 2021). Currently, household and personal care applications are the most prominent sectors for surfactant usage. Market forecasts also indicate that the Asia-Pacific region, particularly China and India, is expected to witness increasing consumption of surfactants (Future et al., 2004). In 2021, China’s surfactant industry revenue reached USD 54.2 billion, and the market for soaps, washing powders, and synthetic detergents is projected to grow by 5% annually. Additionally, the rising disposable income in India may drive consumer preferences towards premium products, further bolstering and boosting the surfactant market in the country (Mordor Intelligence, 2022).

Despite the demand for surfactants is enormous; the production of these petrochemical-derived compounds has accelerated climate change and the depletion of natural resources, posing a significant threat to our planet (Naveenkumar and Baskar, 2021). There is also a growing awareness of the environmental risks and safety issues associated with the disposal of synthetic surfactants, including their toxicity, poor biodegradability, and eutrophication-causing effects. The United Nations (UN) member states adopted the 2030 Agenda for Sustainable Development Goals (SDGs) in 2015, encouraging the use of renewable resources and the means to achieve global sustainability in which the balance between social, economic, and environmental aspects can be achieved (Bautista et al., 2016). Therefore, it is crucial to generate more sustainable and environmentally friendly substitution.

Biosurfactants, a natural product of bacterial or fungal metabolism, are gaining increasing attention (Rebello et al., 2014; Rocha e Silva et al., 2019; Farias et al., 2021). They possess desirable physicochemical properties, biodegradability, biocompatibility, and chemical diversity characteristics (Macaulay and Rees, 2014; Rebello et al., 2020), having a broad market prospect. In 2020, the market was dominated by synthetic surfactants, accounting for a substantial 96% share, while bio-based surfactants only represented 4% (Rebello et al., 2020). Nevertheless, the global bio-based surfactant market is projected to experience remarkable growth, reaching a value of USD 6.4 billion by 2025, with an annual growth rate of 5.5% (Research and Markets, 2023).

The conventional biosurfactant process utilizes a combination of hydrophilic and lipophilic feedstocks to optimize production (To et al., 2022). Consequently, a typical production medium contains pure chemicals such as glucose and oleic acid. Although bioprocessing with refined feedstocks generally results in high biosurfactant titres (Gao et al., 2013), these first-generation feedstocks have relatively high production costs with adverse environmental effects (Baccile et al., 2017). Therefore, researchers have explored the use of second-generation feedstocks, specifically industrial residual biomass waste streams (Gaur et al., 2022b). These sustainable biosurfactants offer an environmentally friendly alternative to synthetic surfactants, contributing to the achievement of several UN SDGs related to industry, innovation, responsible consumption and production, climate action, the preservation of marine and terrestrial life, and partnerships for sustainable development. Moreover, they conform to the principles of circular bioeconomy, which emphasizes maximizing the value of biological resources and minimizing the waste (Agrawal et al., 2023).

In fact, scholars are actively investigating a variety of high-value-added products derived from second-generation feedstocks (primarily biomass), such as poly (3-hydroxybutyrate-co-3-hydroxyvalerate) (PHBV) (Hathi et al., 2022), succinic acid (Li et al., 2019), hydroxymethylfurfural (HMF) (Yu et al., 2018), recombinant protein (Mou et al., 2024), and hydrogels (Kaur et al., 2023a; Kaur et al., 2023b), which is also known as biorefinery. However, the practical application of these waste feedstocks in biorefineries faces challenges due to their inherent heterogeneity. Moreover, the titres generated from second-generation feedstocks such as sunflower oil, waste fried oil, jatropha oil, and animal fat (as hydrophobic substrates), and sugarcane molasses, soy molasses, and glycerol (as hydrophilic substrates) have been relatively low (Hayes et al., 2019; Jimenez et al., 2019). To reduce the production cost of bioproducts, it is necessary to increase the productivity and titres derived from second-generation feedstocks to a level comparable to those obtained from first-generation feedstocks.

This review summarized the applications of biosurfactants and associated products in the market, discussing sustainable production of biosurfactants and secondary feedstocks used in biosurfactants production, and the optimization of biosurfactant production. Final challenges and opportunities in sustainable biosurfactant production are also discussed.

## 2 Classification of biosurfactant molecules

**FIGURE 1:**
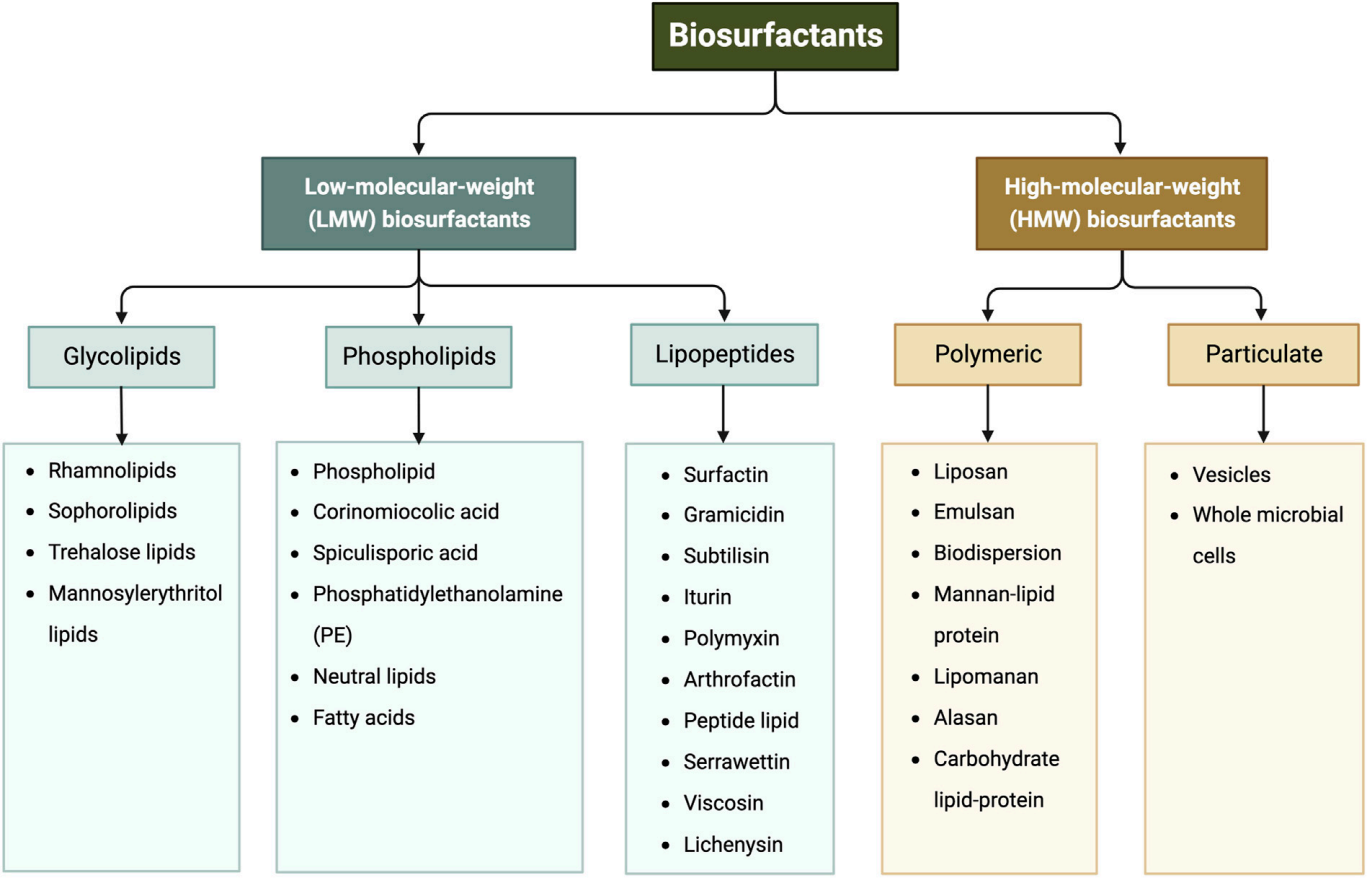
Classification of biosurfactants based on their chemical composition, adapted from (Gayathiri et al., 2022; Mgbechidinma et al., 2022).

**FIGURE 2:**
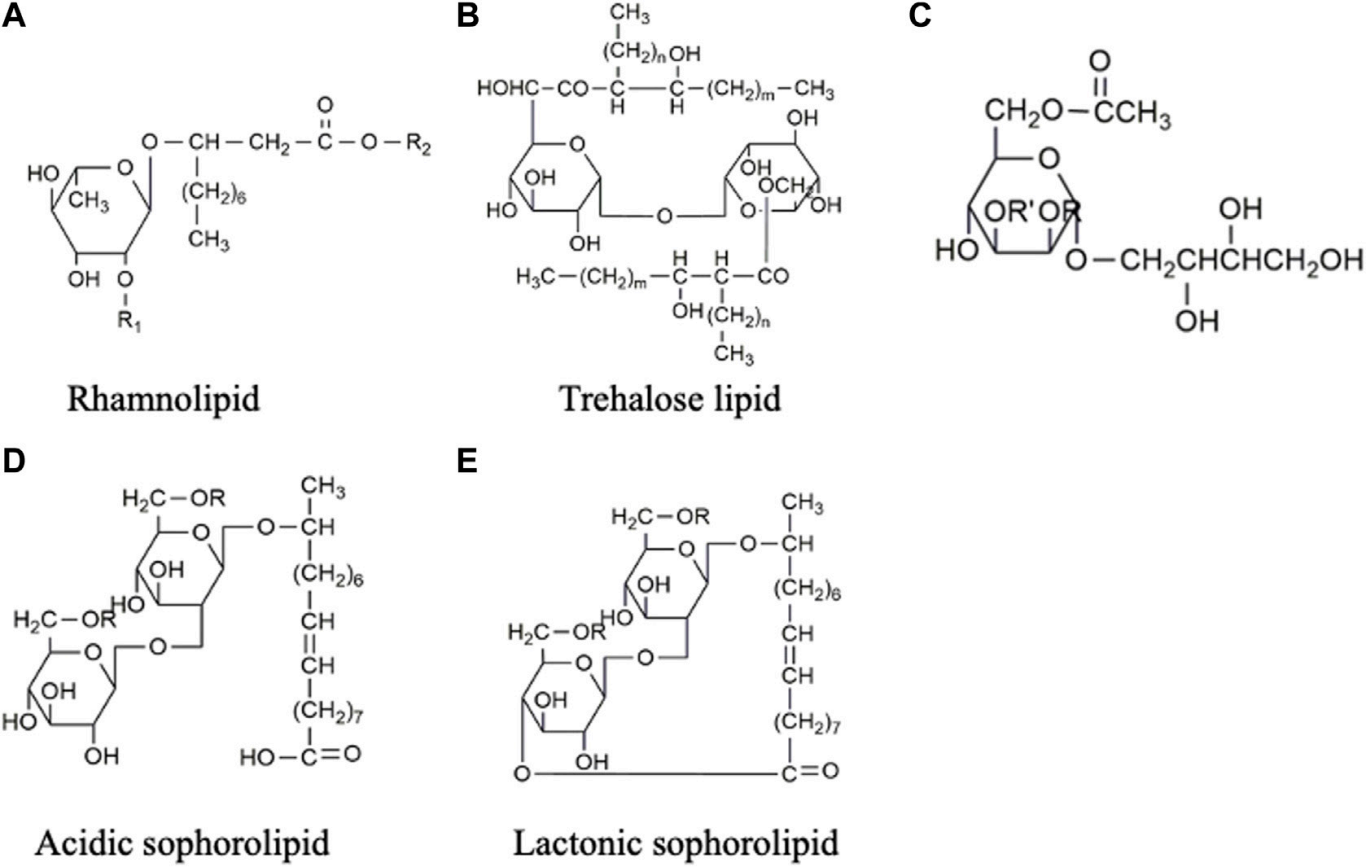
Chemical structures of glycolipids: **(A)** Rhamnolipid, the second rhamnose molecule can be added to R_1_ position to form di-rhamnolipid, and R_2_ represents the attachment of various lengths of fatty acids. **(B)** Trehalose lipid, **(C)** Mannosylerythritol lipid, **(D)** Acidic sophorolipid, and **(E)** Lactonic sophorolipid.

Biosurfactants are a broad group of chemicals with a wide range of molecular structures and functions, making them applicable in various industries. They can be broadly categorized into high- and low-molecular-weight biosurfactants based on their molecular weights, ranging from 500 Da to 1,500 Da (Zuckerberg et al., 1979). Generally, low-molecular-weight (LMW) biosurfactants, such as glycolipids, lipo-peptides and phospholipids, are useful for reducing surface tension at air–water interfaces and interfacial tension at oil–water interface (Rosas-Galvan et al., 2018). On the other hand, high-molecular-weight (HMW) biosurfactants, such as lipoproteins (i.e., complex lipopeptides) and polymeric surfactants, can firmly adhere to various surfaces and act as bio-emulsifiers (Mnif and Ghribi, 2015; Jahan et al., 2020). Figure 1 shows the classification of biosurfactants based on their chemical composition. This review mainly focuses on glycolipids, which consist of one or more hydrophilic carbohydrate moieties linked to hydrophobic fatty acid chain(s) of various lengths via ester bonds (Jahan et al., 2020). Among them, rhamnolipids, trehalolipids, sophorolipids, and mannosylerythritol lipids are selected as the main subjects as they hold the prime market share attributed to high demand from end-use sectors, especially cosmetic and personal care industries (Fortune Business insights, 2022). Figure 2 shows the structures of the four glycolipids.

### 2.1 Rhamnolipids

Rhamnolipids (RLs) are made up of one to two rhamnose sugars linked to up to two molecules of fatty acid chains of 8–16 carbons (Abdel-Mawgoud et al., 2010) (Figure 2A). Rhamnolipids can be produced from certain bacterial species such as *Serratia rubidaea* (Nalini and Parthasarathi, 2018), *Lysinibacillus sphaericus* (Gaur et al., 2019), while the genus *Pseudomonas* is the major producer, including *Pseudomonas aeruginosa* (de Araujo Padilha et al., 2019) and *Pseudomonas cepacian* (Silva et al., 2014). The molecular structure of the rhamnolipid may change depending on several factors, such as culture conditions, carbon sources, and strains used, resulting in variations in surfactant properties. Rhamnolipid produced from *P. aeruginosa* can reduce the surface tension from 72 to 30 mN m^-1^ and interfacial tension from 43 to 1 mN m^-1^, respectively (Christova et al., 2011; Khademolhosseini et al., 2019). The critical micelle concentration (CMC) of the rhamnolipids also varies in chemical composition and structure, which ranges from 50 to 200 mg L^-1^ (Santos et al., 2016; Camara et al., 2019; Khademolhosseini et al., 2019). Recent studies have indicated that rhamnolipid possesses antimicrobial properties against pathogens such as *L. monocytogenes*, *Bacillus cereus,* and *S. aureus* (de Freitas Ferreira et al., 2019). Moreover, they have demonstrated cytotoxic effects on colorectal (Twigg et al., 2022) and breast cancer cells (Rahimi et al., 2019). These findings have sparked ongoing investigations into the potential application of rhamnolipids in the food and pharmaceutical industry.

### 2.2 Trehalose lipids

Trehalose lipids (also known as trehalolipids) can be synthesized by various genera such as *Rhodococcus* (Kuyukina et al., 2015), *Nocardia* (Christova et al., 2015), *Mycobacterium* (Christova et al., 2015), *Gordonia* (Delegan et al., 2020) and *Arthrobacter* (Suzuki et al., 1969). Among these, the *Rhodococcus* genus has been extensively investigated and favoured in industrial production due to its lowest number of pathogenic and opportunistic species (Haburchak et al., 1978). The trehalose lipids (Figure 2B) produced by *Rhodococcus* offered higher structural diversity and appeared to be a mixture of trehalose 6,6-dicorynomycolates with the general formula of C_186_H_366_O_17±10_ CH_2_ (Noll et al., 1956). A typical trehalose lipid produced from *Rhhodococcus erythropolis* can lower surface and interfacial tensions to a range of 25–40 mN m^-1^ and 1–5 mN m^-1^, respectively (Santos et al., 2016).

### 2.3 Mannosylerythritol lipids (MEL)

Mannosylerythritol lipids (MEL) are produced from fungal genera, such as *Ustilago, Pseudozyma, Kurtzmanomyces, Schizonella*, and *Moesziomyces* (Jahan et al., 2020). In the production process, vegetable oil such as soybean oil (SBO) or rapeseed oil (RO) are commonly used as substrates, resulting in high titres exceeding 50 g L^-1^ (Nascimento et al., 2022). The structure of MEL, as shown in Figure 2C, consists of mannose sugar connected to the fatty acid chain. MEL can be further subclassified according to the degree of mannose acetylation, ranging from non-acetylated (MEL-D) to monoacetylated (MEL-B/MEL-C) and diacetylated (MEL-A) forms (Sałek and Euston, 2019). MEL derivatives are capable of lowering surface tension to 72—31 mN m^-1^, making them suitable for stabilizing water/oil emulsions in the cosmetic industry (Recke et al., 2013). Among the different homologues (-A, -B, -C, and -D), MEL-B stands out in the skincare field (de Andrade et al., 2022). Several companies, including DKBIO (Daejeon, Korea) (Tran et al., 2022) and Toyobo (Osaka, Japan) (Saika et al., 2016), have successfully commercialized the submerged fermentation process for MEL production in the cosmetic industry.

### 2.4 Sophorolipids (SLs)

Sophorolipids (SLs) comprise a hydrophilic and a hydrophobic moiety (Figures 2D,E). The hydrophilic end is formed by a disaccharide sophorose, connected through a β-1, 2 bond. The hydrophobic moiety is composed of a hydroxylated fatty acid with a chain length of C_16_ or C_18_, which can be either acetylated or non-acetylated. These moieties are held together by glycosidic bonds (Qazi et al., 2022). SLs are extracellularly produced by yeasts such as *Candida apicola* (Gorin et al., 1961), *Torulopsis magnolia* (Hommel and Huse, 1993), *Rhodotorula bogoriesis* (Nunez et al., 2004) and *Starmerella bombicola* (Solaiman et al., 2007). A typical SLs production results in a mixture of acidic and lactonic SLs. In acidic SL (Figure 2D), the carboxylic end of the fatty acid is liberated, while in lactonic SL (Figure 2E), the carboxylic end is esterified at the C_4_’, C_6_’ or C_6_″ position, forming a ring structure (Celligoi et al., 2020). The different molecular structures of lactonic SL and acidic SL contribute to their distinct properties. Lactonic SL is more effective in reducing surface tension and possesses higher antimicrobial properties, while acidic SL has better foam-forming capabilities (Kwak et al., 2021). On the other hand, the increase in acetylation of the fatty acid chains decreases the hydrophilicity of SLs and improves their antiviral properties (Lydon et al., 2017). Although SLs can be produced by different strains, *Starmerella bombicola* is considered the most productive strain with a maximum titre of 300 g L^-1^ (Wang et al., 2019), which makes SLs have progressed furthest in the biosurfactant industry for commercial applications.

## 3 Applications of glycolipid biosurfactants

**FIGURE 3:**
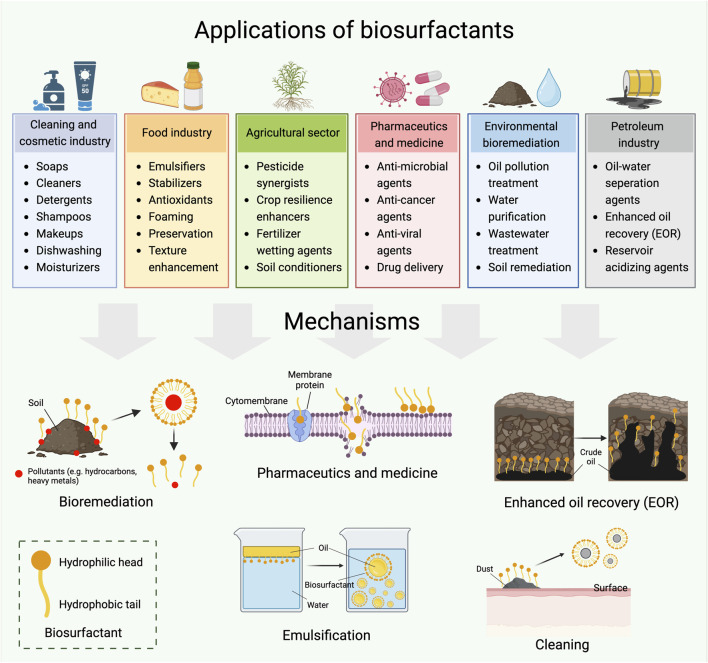
Potential applications of biosurfactants and their mechanisms.

The application of biosurfactants is much broader than that of their fossil based counterparts. In addition to their fundamental applications in the cleaning and personal care industries, biosurfactants have a wide range of other application areas (Ambaye et al., 2021). They can be used as emulsifiers in the food industry; medicines, moisturizers and creams in the pharmaceutical industry; fertilizers in the agricultural sector; for waste and sewage treatment in civil waste industries; and for oil extraction and bioremediation in the petroleum industry (Ribeiro et al., 2020a). Figure 3 provides an overview of potential biosurfactants applications and their mechanisms. In this review, glycolipids are selected as model biosurfactants, and their potential applications in various fields are investigated.

### 3.1 Glycolipid biosurfactants used for bioremediation

#### 3.1.1 Bioremediation of hydrocarbon contaminants

Pollution from heavy metals and hydrocarbons negatively affects the ecosystems and human health, thereby hindering the UN-SDGs (Sonowal et al., 2022). Hydrocarbon pollutants encompass alkanes, aromatic compounds, chlorinated hydrocarbons, heterocyclic nitrogen, and nitroaromatic compounds (Effendi et al., 2017). Traditional methods of separating hydrocarbons without chemical alteration have proven ineffective due to their strong adhesion to the soil matrix. In contrast, biological processes offer efficient and cost-effective remediation methods.

The biodegradation of polycyclic aromatic hydrocarbons (PAHs) faces limitations due to their extreme hydrophobicity and poor solubility. Nevertheless, biosurfactants play a crucial role in enhancing the bioavailability of hydrophobic pollutants to microbes, promoting their biodegradation by facilitating the solubilization and mass transfer of PAHs into microbial cells (Zhang et al., 2015; Effendi et al., 2018; Kreling et al., 2020). For example, a glycolipid produced by *P. aeruginosa* strain S5 was found to effectively facilitate the biodegradation of high-molecular-weight PAHs in cooking wastewater, significantly reducing their concentration from 9,141.02 to 5,117.16 μg L^-1^ within 15 days (Sun et al., 2019). The biosurfactant produced by the strain S5 reduced the surface tension from 72.2 to 29.6 mN m^-1^ and exhibited a relatively lower CMC value of 96.5 mg L^-1^, which is far lower than that of synthetic surfactants (Liang et al., 2014; Wang et al., 2018). Moreover, the strain S5 biosurfactant displayed high stability even in slightly acidic and alkaline environments, along with a broad tolerance to NaCl concentrations ranging from 0% to 15%, making it suitable for diverse environmental applications (Wang et al., 2018). Similarly, a potent glycolipid from *Streptomyces* sp. SN JASM6 can utilize hydrocarbons as energy sources for biosurfactant production, exhibiting a new possibility for efficient biodegradation of hydrocarbons (Javee et al., 2020).

#### 3.1.2 Bioremediation of contaminated soil

Biosurfactants have demonstrated their potential in the remediation of contaminated soil. Haloi and Medhi (2019) isolated *Achromobacter* sp. TMB1 from soils near local petrol pumps and identified 10 different types of mono- and di-rhamnolipids congeners with the fatty acids carbon length ranging from C_8_ to C_12_ from this strain. Further experiments showed the stability of these biosurfactants within a temperature range of 20°C–100°C and a pH range of 2–12, maintaining the structural integrity up to 550°C, indicating their potential in bioremediation (Haloi and Medhi, 2019). In a recent study, Yang et al. (2023) introduced sophorolipids and rhamnolipids into petroleum-contaminated soil to facilitate the bioremediation of pollutants by *Gordonia alkanivorans* W33. By mixing the bacteria with sophorolipids and rhamnolipids in a weight ratio of 9:10, significant degradation of the petroleum in the soil was observed. Approximately 56.3% of the petroleum with 20,000 mg/kg petroleum content was degraded, with an average degradation rate of 250.2 mg d^-1^ (Yang et al., 2023). The mixture of sophorolipid and rhamnolipid is believed to improve the ability to form microemulsions from wide range of hydrocarbon (Nguyen and Sabatini, 2011). Zhang et al. (2022b) conducted a study on the remediation of soil co-contaminated with phenanthrene (PHE) and cadmium (Cd) using biosurfactant-enhanced soil washing. The presence of both contaminants resulted in changes to the soil structure and rhamnolipid micelle, leading to different removal rates compared to soils contaminated with only one substance. Results indicated that PHE was effectively captured within the micelles of rhamnolipid, while Cd formed complexation with the external carboxyl groups of the rhamnolipid micelle. By optimizing the conditions, the removal rates of Cd and PHE reached 72.4% and 87.8%, respectively (Zhang et al., 2022b).

#### 3.1.3 Bioremediation of heavy metals

The accumulation of heavy metals in living organisms poses a significant concern for food safety and human health (Taha et al., 2010; Hou et al., 2020). Among these metals, chromium (Cr) ranks as one of the most toxic pollutants globally. Various technologies, such as soil removal, soil washing, stabilization, and flushing, have been developed to address this issue, but they lack reliability and cost-effectiveness (Mulligan, 2009). On the contrary, using microorganisms in bioremediation offers a more sustainable approach, where biosurfactants can facilitate the biosorption by forming complexes with free metals in the solution or establishing point contact with the metals at the solute-solvent interface under conditions of low surface tension (Kamarudheen et al., 2020). A study by Arisah et al. (2021) demonstrated the effectiveness of *P. aeruginosa* RW 9 in reducing the Cr VI) concentration. The presence of Cr VI) ions stimulated the secretion of rhamnolipid by the microorganisms, which helped with bioremediation. The bioremediation of Cr VI) by *P. aeruginosa* RW 9 occurred in two distinct ways. Firstly, rhamnolipid acted as a protective barrier, preventing the hazardous ions from penetrating the cells. Secondly, Cr VI) was transformed into a less harmful form, Cr III), through the formation of complexes. As a result, this strain removed 85% of Cr VI) when the initial concentration was 10 mg L^-1^ within 4 h (Arisah et al., 2021). Poirier et al. (2023) proposed a new mechanism using metallogelation properties of the biosurfactant to remove heavy metals from water. The formed compound is able to complex cations in water, thus trapping heavy metals in the gel phase. This mechanism allows the removal of up to 95% for cobalt (Co^2+^) and 88 ± 10%, 80 ± 3%, and 59 ± 6% for copper (Cu^2+^), nickel (Ni^2+^), and Cr^2+^, respectively (Poirier et al., 2023). Besides, the removal of other heavy metals by biosurfactants, including Cu (Li et al., 2017), zinc (Zn) (da Rocha Junior et al., 2019), cadmium (Cd) (Huang and Liu, 2013), lead (Pb) (Sun et al., 2021c), and iron (Fe) (Luna et al., 2016) were also observed in various studies.

### 3.2 Glycolipid biosurfactants used in the food industry

#### 3.2.1 Improving food characteristics

Microbial surfactants offer a versatile means to enhance various food properties, including emulsification, foaming, thickening, texture enhancement, and preservation. For example, rhamnolipids have been recognized for improving bakery products by enhancing attributes such as dough volume, shape and stability (Sharma, 2016). Furthermore, a study conducted by Ribeiro et al. (2020b) explored the utilization of biosurfactants from *Saccharomyces cerevisiae* URM 6670 as a substitute for egg yolk in cookie formulation, with no observable alterations to the physical or physicochemical characteristics of the dough. The incorporation of biosurfactants led to cookies with increased levels of linoleic acid (C_18:2_), which is a source of beneficial polyunsaturated fatty acids (PUFAs) known for their potential to mitigate cardiovascular disease (Ribeiro et al., 2020b).

#### 3.2.2 Preventing food spoilage

A significant role of biosurfactants in the food industry is safeguarding against food spoilage due to their inherent antiadhesive and antimicrobial attributes. Their biodegradable, non-toxic, and stable nature in different environmental conditions, including temperature, pH, and salinity, makes them suitable for various applications such as food surface cleaning, packaging, coating, transportation, and storage processes (Ribeiro et al., 2020a; Nalini et al., 2020).

Considering the risk of food contamination during food preparation, the pathogen *Listeria monocytogenes* is particularly notorious for causing severe illness. Sun et al. (2021b) investigated the inhibitory effects of two commercial glycolipid products, Nagardo™ and rhamnolipids, against *L. monocytogenes* in milk and cheese. Their findings indicated that rhamnolipids caused undesirable colour changes and coagulation in whole milk, while Nagardo™ was more suitable for addition to milk at a concentration below 1100 mg L^-1^. Nagardo™ exhibited minimum inhibitory concentration (MIC) and minimum bactericidal concentration (MBC) values of 800 and 1100 mg L^-1^, respectively. Notably, Nagardo™ demonstrated more potent antibacterial effects in skimmed milk, significantly reducing cell counts at a concentration of 1,000 mg L^-1^ (Sun et al., 2021b).

Moreover, glycolipids have demonstrated effectiveness in inhibiting spore germination. Sun et al. (2021a) assessed the impact on spore-forming strains such as *Paenibacillus odorifer*, *Bacillus weihenstephanensis*, and *Viridibacillus arenosi*, which are known to survive pasteurization and cause spoilage during refrigerated storage. Their findings revealed that higher concentrations (400 mg L^-1^) of glycolipids were necessary to inhibit *Viridibacillus arenosi* spore germination, while concentrations of 400 and 200 mg L^-1^ significantly impeded the outgrowth of *P. odorifer* and *B. weihenstephanensis* in whole milk. This suggests that introducing glycolipids at a concentration of 400 mg L^-1^ to whole milk could potentially prevent spoilage caused by spore-forming bacteria (Sun et al., 2021a).

### 3.3 Glycolipid biosurfactants used in the petroleum industry

#### 3.3.1 Enhanced oil recovery

Currently, the petroleum industry continues to play a significant role in energy production, and the efficiency of crude oil extraction remains a crucial factor. The process of oil production can be divided into three stages. Initially, primary production involves extracting roughly 10%–20% of crude oil through natural pressure. However, as the pressure decreases, the crude oil yield also diminishes. To counter this, the second stage employs the injection of water or air into the oil reservoir, thereby enhancing the crude oil yield to a range of 40%–50%. Subsequently, chemical or thermal methods are employed in the tertiary stage to further augment the extraction rate. These methods encompass the utilization of hydrocarbon solvents, synthetic surfactants, gases, or combinations to reduce the surface tension between the oil and water interfaces (Thomas, 2008). While these chemical techniques enhance oil recovery, they unfortunately contribute to environmental pollution. Considering this, microbial enhanced oil recovery (MEOR) has emerged as a more ecologically friendly and cost-effective alternative for tertiary recovery (Gudina et al., 2012). This approach involves the use of microorganisms to produce biosurfactants, which aid in recovering oil by releasing it from rock formations. MEOR can be executed through three methods (Al-Bahry et al., 2013; Bachmann et al., 2014): direct injection of biosurfactants into the reservoir, introduction of biosurfactant-producing microorganisms into the reservoir, or introduction of nutrients to encourage the growth of indigenous biosurfactant-producing microorganisms.

Recent research has focused on the application of glycolipid biosurfactants to improve oil recovery. Specifically, the effectiveness of rhamnolipids and sophorolipids in separating crude oil from water has been explored. Under optimal concentrations and salinities, rhamnolipids and sophorolipids have demonstrated ORR of 70% and 61%, respectively, in a heterogeneous micromodel that simulates carbonate reservoirs in southern Iran (Aghaei et al., 2023). In another study, the use of sophorolipids effectively separated oil from oily sludge, resulting in a remarkable recovery rate of 78.62% of crude oil from the sludge. Furthermore, researchers have integrated nanoparticles into the process to further enhance oil recovery. By combining rhamnolipids and sophorolipids with silica nanoparticles, a reduction in both surface tension and interfacial tension was observed, leading to an additional ORR of 20% and 25% for sophorolipids and rhamnolipids, respectively, with an optimal concentration of 40 ppm of silica nanoparticles (Joshi et al., 2022).

#### 3.3.2 Oil pollution remediation

Recently, increased attention has been directed toward isolating and identifying microorganisms that produce biosurfactants or have the capacity to degrade oil in oil-fields. For instance, *Bacillus siamensis*, isolated from an oilfield in northwest China, has demonstrated the ability to degrade various fractions of heavy oil and enhance MEOR (Zhang et al., 2022a). Similarly, *Franconibacter* sp. IITDAS19, isolated from crude oil-contaminated soil in the Lakwa oil field, India, has been identified as a producer of rhamnolipids and di-rhamnolipids, achieving a maximum MEOR of 63% ± 4.2% (Sharma et al., 2022). *Pseudomonas* sp. TMB*2* and *Achromobacter* sp. TMB1, also isolated from contaminated soil, have been found to produce mono-rhamnolipids and di-rhamnolipids. *Pseudomonas* sp. TMB2 exhibited a 16.7% MEOR effect, and *Achromobacter* sp. TMB1 proved effective in removing total petroleum hydrocarbons from sludge at a rate of 90.12% ± 1.2% (Haloi and Medhi, 2019; Haloi et al., 2020).

The glycolipid-producing strains identified in hydrocarbon-contaminated environments are mainly bacteria. However, only a limited number of fungal strains, particularly from the *Aspergillus* genera, are known to produce glycolipids and lipopeptides. *Aspergillus fumigatus* Shu2, recently isolated in the oil refining industry in Malaysia, has demonstrated the ability to produce biosurfactants and degrade hydrocarbons simultaneously. Notably, this strain achieved a degradation rate of 57% ± 2% for total petroleum hydrocarbons in 16 h, which further improved to 63% ± 2% with the addition of nutrients (Othman et al., 2022).

### 3.4 Biomedical functions of glycolipid biosurfactants

#### 3.4.1 Antimicrobial activity

The antimicrobial activity of glycolipids involves both direct antimicrobial action and the inhibition of biofilm formation (Ng et al., 2023). Biofilms, which consist of surface-associated bacterial cells, enable bacteria to survive in adverse conditions and contribute to antibiotic resistance through mechanisms like growth rate adjustment, antimicrobial resistance neutralization, and gene expression (Ciofu et al., 2022). Patel et al. (2021) isolated *Lactobacillus rhamnosus* from human breast milk, which can produce glycolipid-type biosurfactants with antibacterial properties against several pathogens. The derived glycolipid demonstrated MIC and MBC values ranging from 12.5 to 50 mg mL^-1^ and 25–1000 mg mL^-1^, respectively, against pathogens such as *Bacillus subtilis*, *P. aeruginosa*, *S. aureus*, and *E. coli* (Patel et al., 2021). This glycolipid was found to impede bacterial adhesion and biofilm formation by promoting microbial cell detachment through sloughing, erosion, and abrasion. It also interfered with quorum sensing signalling, disrupting biofilm development, motility, and pathogenicity (Quadriya et al., 2018). In another study, the glycolipid derived from *Bacillus licheniformis* SV1 effectively combats the formation of the *Candida glabrata* biofilm at a concentration of 1 mg mL^-1^. This glycolipid induces cell death by elevating the production of reactive oxygen species (ROS) (Gupta et al., 2021). Manikkasundaram et al. (2023) demonstrated a biosurfactant from *Streptomyces* sp. HRB1 inhibits biofilm formation in *P. aeruginosa* and quorum sensing in *Chromobacterium violaceum* MTCC 2656. Additionally, this biosurfactant exhibited anti-proliferation effects against leukaemia and myeloma (Manikkasundaram et al., 2023). Similarly, a glycolipid derived from *Shewanella algae* exhibited considerable growth inhibition of clinical bacterial pathogens and disrupted the preformed biofilms of *B. cereus* (83%), *S. pneumoniae* (53%), *P. aeruginosa* (92%), *E. coli* (64%), *K. pneumoniae* (87%), and *Acinetobacter* sp. (72%) (Gharaei et al., 2022).

#### 3.4.2 Anticancer and antiviral activity

Researches have been conducted on the anticancer properties of various glycolipids, including lactonic-sophorolipid, acidic-sophorolipid, glucolipid, and bolalipid, on different cancer cell lines (Haque et al., 2021). Results indicated that lactonic sophorolipid and glucolipid induce the generation of ROS, disrupt mitochondrial membrane potential, and ultimately trigger necrotic cell death. Furthermore, a synergistic anticancer effect was observed when combining lactonic sophorolipid and glucolipid in the A549 cell line. In the context of melanoma, a particularly aggressive form of skin cancer, glycolipids demonstrated cytotoxicity against murine melanoma cells, while having lesser effects on fibroblasts and human erythrocytes. Such cytotoxicity was attributed to apoptosis induction mediated by nitric oxide-triggered reactive oxygen species generation (Feuser et al., 2021a). Different types of sophorolipids and rhamnolipids were studied for their impact on normal human keratinocytes (HaCaT) and malignant melanocytes (SK-MEL-28). Lactonic sophorolipids and mono-rhamnolipids displayed selective inhibitory effects, significantly impairing melanoma cell viability at a concentration of 40 μg mL^−1^, compared to a higher concentration of 60 μg mL^−1^ for healthy human keratinocytes. Necrosis was identified as the primary mechanism of cell death induced by these glycolipids (Feuser et al., 2021a; Adu et al., 2022).

Furthermore, glycolipids have been identified to possess antiviral effects against SARS-CoV-2 and COVID-19. Specifically, sophorolipids can solubilise the lipid envelope of SARS-CoV-2, rendering the virus inactive. These sophorolipids also function as immunomodulators, mitigating the cytokine storm induced by SARS-CoV-2 and halting the progression of COVID-19 in patients (Daverey et al., 2021).

#### 3.4.3 Enhancing drug delivery

Moreover, glycolipids demonstrate potential in vaccine development, drug delivery systems, and combination therapies. They show promising potential as vaccine adjuvants by stimulating natural killer T (NKT) cells, thus activating antigen-presenting cells and promoting an inflammatory cytokine response. This process enhances the CD8^+^ T-cell response against tumour-associated peptides and helps prevent breast cancer metastasis to the lungs (Burn et al., 2022). Additionally, glycolipid-based polymeric micelles (GLPM) have been used to encapsulate an angiotensin II receptor I inhibitor (telmisartan) and a cytotoxic drug (doxorubicin), facilitating drug delivery and enhancing the antitumor effect through the peroxisome proliferator-activated receptor-gamma pathway (Zhu et al., 2019). Furthermore, glycolipids conjugate modified with the lipophilic agent IR-780 iodide (CSOSA) were employed as delivery carriers to enhance the efficacy of chemo-phototherapy, particularly with doxorubicin (DOX). This approach improved drug delivery and accumulation in mitochondria, resulting in elevated ROS formation and cell death (Tan et al., 2019).

### 3.5 Glycolipid biosurfactants used in agriculture

#### 3.5.1 Applications in pesticide industries

Biosurfactants can act as adjuvant with herbicides, biopesticides, fungicides, and anti-zoospore agents (Naughton et al., 2019). For example, Mannosylerythritol lipids (MEL) of subtype -A and -B have demonstrated potent inhibition against the germination of the pathogen *Blumeria graminis f. sp. tritici* T-10 which causes wheat powdery mildew (Yoshida et al., 2015). Additionally, mono-rhamnolipids and di-rhamnolipids from *P. aeruginosa* A4 have been found to inhibit the growth of *Aspergillus flavus* F2, *Aspergillus niger* F14, *Cunninghamella bertholletiae* F1 and *Rhizopus oryzae* F5, by 54%, 61%, 59% and 50%, respectively. Those strains have been identified to cause root rot in palm seeds. Besides, rhamnolipids were found to be more effective against the spore germination of *A. flavus* F2 and *R. oryzae* F5 at MIC concentrations as low as 2.75 mg mL^-1^, indicating its high antifungal activity (Onlamool et al., 2023).

*Xanthomonas oryzae* is one of the most devastating diseases in rice worldwide, particularly in Asia, where glycolipid-type biosurfactants from endophytic *Acinetobacter* sp. ACMS25 were found to reduce the growth rate and population of *X. oryzae* by 38.4% and 43.5%, respectively. Additionally, these biosurfactants have demonstrated the ability to improve germination and provide protection against the disease (Shalini et al., 2017). Benson et al. (2022) showcased the use of biosurfactants from *Achromobacter xylosoxidans* AUM54 together with its biosurfactant to suppress *Ralstonia solanacearum* growth, which causes bacterial wilt in tomatoes. This biosurfactant prompted the production of disease-related enzymes, such as phenylalanine ammonia-lyase, polyphenol oxidase, and peroxidase, ultimately reducing the infection (Benson et al., 2022). Similarly, antifungal effects have been observed against *Phytophthora infestans* by reducing the lesion area of late blight disease at 0.2% concentration in 5 days using biosurfactants from *P. aeruginosa* PA1. However, it should be noted that although this biosurfactant contains mono-rhamno-di-lipidic that is effective against late blight disease, the high concentrations of these biosurfactants (0.3%v v^−1^) can lead to slight phytotoxicity (Tomar et al., 2019).

#### 3.5.2 Promoting plant growth

In agriculture, biosurfactants can enhance the nutrient supply to beneficial microorganisms linked to plants, which also exhibit antimicrobial effects against plant pathogens (Cameotra et al., 2010). These biosurfactants also augment the availability of naturally occurring micronutrients. For example, plant-growth-promoting rhizobacteria (Bp-PGPR) strains like *B. subtilis* and *P. fluorescens* can help plants acquire resources like nitrogen and phosphorus, thus fostering plant growth directly and indirectly (Chopra et al., 2020; Kumar et al., 2021). Similarly, glycolipids from endophytic *Bacillus pumilus* 2A enhanced the growth of *Phaseolus vulgaris L.* (bean), *Raphanus L.* (radish) and *Beta vulgaris L.* (beetroot) at 0.2% concentration. The microbial surfactants might indirectly facilitate plant growth by enhancing the bioavailability of hydrophobic compounds to microorganisms within the rhizosphere (Marchut-Mikołajczyk et al., 2021).

Another strain, *Pseudomonas guariconensis* LE3, not only produces mono- and di-rhamnolipids with antagonistic properties against *Macrophomina phaseolina* (causing charcoal rot of sunflowers) but also synthesizes antibiotics such as diacetylphloroglucinol, phenazine 1-carboxylic acid and pyocyanin. Additionally, it produces lytic enzymes like chitinase and endoglucanase, exhibiting broad-spectrum antagonistic activity against both fungi and bacteria. Furthermore, the author also found that the biosurfactant obtained from the strain LE3 effectively increased the root adherence to soil and resulted in a substantial increase in crop yield (80.80%) and biocontrol activity (75.45%). The authors also claimed that the bio-stimulant and biocontrol response were ascribed to selectively inhibiting the plant pathogens and protecting the beneficial microbes from desiccation and death (Khare and Arora, 2021).

### 3.6 Limitations of large-scale biosurfactants application

Although glycolipid biosurfactants have demonstrated promising potential in various sectors, most of the research are still in the bench-scale level. The widespread implementation of biosurfactants at a large scale is still constrained by various factors. Further efforts are required to ensure their effectiveness and the resilience under complex conditions in real-world environments. For example, the complicated soil and weather conditions, as well as the interactions with other microorganisms and contaminants, should be considered when they are applied in bioremediation, petroleum and agriculture sectors (Figure 3). Similarly, the safety, stability, and compatibility of biosurfactants in the food and pharmaceutical industries should be thoroughly examined (Figure 3).

To advance the application of biosurfactants, a detailed understanding of their capabilities and interactions is crucial. Additionally, exploring metabolic engineering techniques to enhance their resistance and adaptation mechanisms can provide a solid framework for their use in real-world matrices. Furthermore, the production costs must be decreased to make them economically viable before they can be widely used.

## 4 Opportunity and challenge of biosurfactants production

### 4.1 Market opportunities of biosurfactants

Petrochemical-based surfactants are being produced in bulk with mature processes. However, they are unsustainable and incompatible with the UN’s SDGs, and concerns have been raised about their toxicity, biocompatibility, and negative impact on the ecosystem. In recent decades, government strategies and market factors have led to the generation of more products consistent with sustainable concepts. Biosurfactants, which are natural products derived from bacterial or fungal fermentation, are considered promising alternatives to traditional synthetic surfactants. Biosurfactants offer better properties, such as low toxicity, improved biocompatibility, and greater environmental friendliness and sustainability (Rebello et al., 2014; Farias et al., 2021). Moreover, replacing fossil-derived surfactants with biosurfactants can reduce CO_2_ emissions by 8%, equivalent to 1.5 × 10^6^ t of CO_2_ emission (Rocha e Silva et al., 2019; Farias et al., 2021). This substantial reduction in emissions positions biosurfactants highly competitive within the context of carbon neutrality, contributing to SDG 13 - Climate Action.

Table 1 summarises the highest reported productivities for different biosurfactants. Among the glycolipids, rhamnolipids can achieve a high productivity of 1.54 g L^-1^h^-1^ by sequential fed-batch fermentation with high cell densities using corn oil and NaNO_3_ as subtracts (Jiang et al., 2021). But when turning to waste stream feedstocks, the rhamnolipids productivity is always lower than 1 g L^-1^h^-1^ (Table 3; Table 4) (Sharma et al., 2019; Pathania and Jana et al., 2020; Baskaran et al., 2021; Gaur et al., 2022a). On the other hand, the remarkable sophorolipids productivity of up to 3.7 g L^-1^h^-1^ in synthetic medium (Gao et al., 2013) has captured significant attention from both the academic and industrial communities, elevating the appeal of sophorolipids. Despite the potential decrease in sophorolipids yields when utilizing waste streams as substrates, a breakthrough study conducted by Wang et al. (2020a) showcased an impressive productivity of 2.4 g L^-1^h^-1^ using food waste as a substrate. Fed-batch fermentation with an *in situ* separation strategy was combined, which allowed high biomass concentration and prolonged fermentation states, resulting in high volumetric productivity (Wang et al., 2020a). This achievement has laid a robust foundation for the commercial production of sophorolipids, reinforcing its viability and potential in the market. However, the productivity of mannosylerythritol lipids and trehalose lipids is relatively lower, even when using the synthetic medium, with the maximum productivity reaching only 0.59 g L^1^h^1^ and 0.21 g L^-1^h^-1^, respectively (Patil et al., 2018; Yu et al., 2022).

**TABLE 1:** The type of glycolipids and their corresponding number of publications based on Scopus database from 2000 to 15 October 2023, and the highest productivity reported.

Glycolipids	No. of research (Scopus search result)	The highest productivity reported (g L^-1^h^-1^)	References^a^
Rhamnolipids	5393	1.54	Jiang et al. (2021)
Sophorolipids	1674	3.7	Gao et al. (2013)
Mannosylerythritol lipids	1155	0.59	Yu et al. (2022)
Trehalose lipids	892	0.21	Patil et al., 2018

^a^ References are related to the reported highest productivity.

**FIGURE 4:**
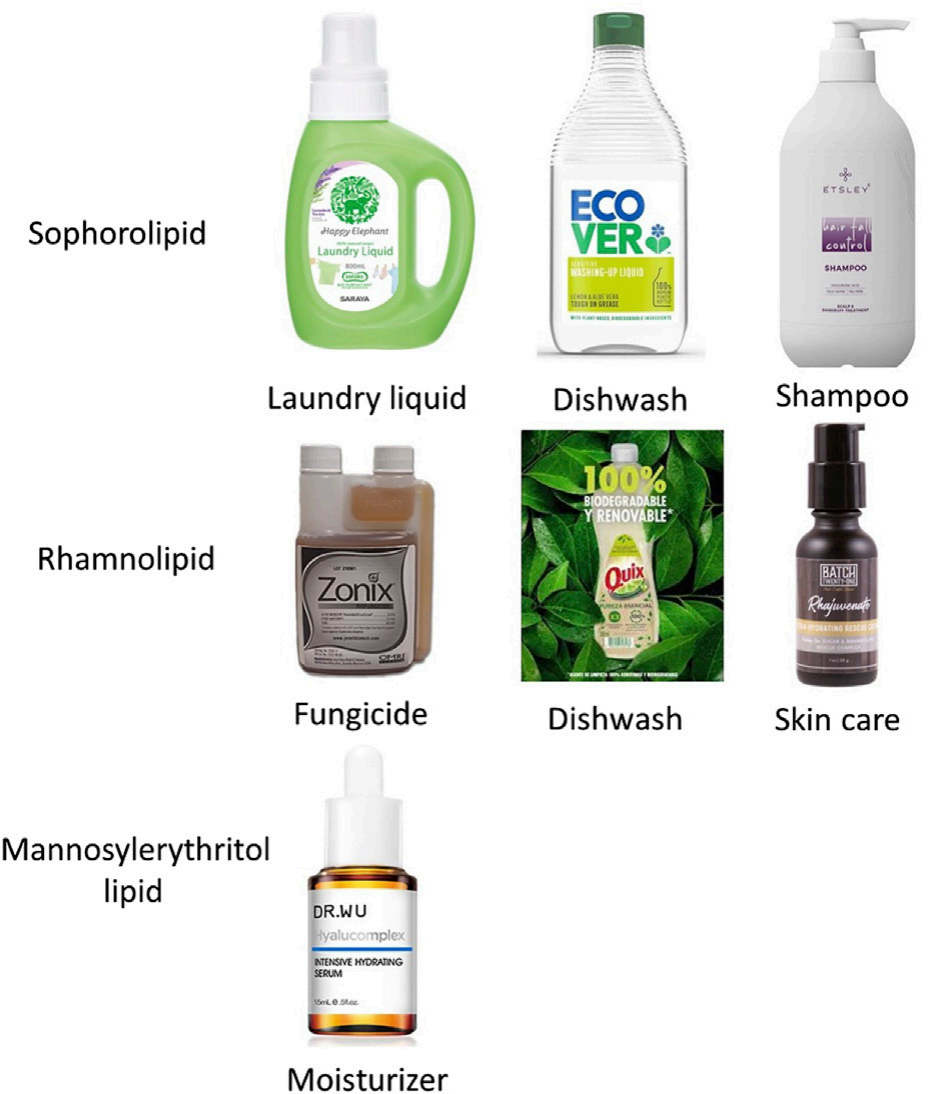
Types of biosurfactant products.

High productivity is crucial to industrial biosurfactant production, leading to extensive research on sophorolipids and rhamnolipids production. On the other hand, rhamnolipids were discovered earlier than sophorolipids (rhamnolipids were first discovered in 1946, while sophorolipids were originally discovered in 1961), which accounts for the greater amount of research focused on rhamnolipid production (Cho et al., 2022; Ashby et al., 2023). As shown in Table 1, a literature search on Scopus, performed on 15 October 2023, using the keywords ‘biosurfactant’ and ‘rhamnolipids’ or ‘sophorolipids’ returned more results compared to a search using ‘trehalose lipids’ and ‘mannosylerythritol lipids’. Table 2 provides an overview of biosurfactants produced by different companies worldwide, indicating that sophorolipids and rhamnolipids are the most popular candidates for biosurfactant production. For instance, several major companies, such as Evonik and Jeneil Biosurfactants, are renowned in the biosurfactant field for producing crude sophorolipid and rhamnolipid products. Kanebo Cosmetics Inc., a Japanese company, has started to produce mannosylerythritol lipids for skin care products. Figure 4 shows the different types of biosurfactant products, including liquid laundry detergents, dishwashing detergents, shampoos, and skin care products. The biosurfactant compositions in these products typically range from 0.01% to 20%, depending on the specific type and functionality (Furuta et al., 2004).

**TABLE 2:** The type of biosurfactants produced by different companies on a commercial scale.

Location	Company	Biosurfactant	Application	Sources
*Asia*				
China	Shanghai Fine Chemical Co., Ltd.	Alkyl polyglucoside APG^®^, GreenAPG is a nonionic surfactant made from renewable vegetal raw material	Used in formulations for household cleaners and personal care.	Shanghai Fine Chemical Co., Ltd.
Japan	Saraya Co. Ltd.	Sophorolipids (Sophoron, a low-foam dishwasher detergent)	Cleaning products, hygiene products	Saraya Co. Ltd
	Allied Carbon Solutions Ltd	Sophorolipids (ACS-Sophor-first bio-based surfactant from Indian mahua oil)	Agricultural products, ecological research	Allied Carbon Solutions Ltd
	Kaneka Co.	Sophorolipids	Cosmetics and toiletry products	Kaneka Co.
North America	AGAE Technologies LLC	Rhamnolipids (R95, an HPLC/MS grade rhamnolipid)	Pharmaceutical, cosmeceutical, cosmetics, personal care, bioremediation (*in situ* and *ex-situ*), Enhanced oil recovery (EOR)	Agaetech LLC
	BOC Sciences Co.	Biosurfactants	Provide services for fermentation production of biosurfactants	BOC science Co.
	Jeneil Biosurfactant Co. LLC	Rhamnolipids (ZONIX, a bio-fungicide and RECO, a rhamnolipid used in cleaning and recovering oil from storage tanks)	Cleaning products, EOR	JENEIL biotechnology
	Paradigm Biomedical Inc.	Rhamnolipids	Pharmaceutical applications	Patents.justia.com
	CD BioGlyco Co.	Rhamnolipids	Service for Custom Rhamnolipid Synthesis	CD Bioglyco Co.
*Europe*				
Belgium	Ecover Belgium	Sophorolipids	Cleaning products, cosmetics, bioremediation, pest control, pharmaceuticals	ecover. com
Germany	BASF SE Co.	BioToLife™ (contains a novel sophorolipid-based ingredient), other glycolipids	Personal Care, Home Care and Industrial Formulators	basf.com
	Fraunhofer IGB	Glycolipids, Cellobiose lipids, MELs	Cleansing products, shower gels, shampoos, washing-up liquids, pharmaceutical (bioactive properties)	igb.fraunhofer.com
UK	TeeGee Biotech	Rhamnolipids and lipopeptides	Pharmaceuticals, cosmetics, antimicrobials and anti-cancer	teegene.co.uk

### 4.2 Production costs and challenges of scaling up

Despite their advantages over non-biological counterparts, the market share of biosurfactants remains relatively low, accounting for only approximately 4% of total surfactant production (Rebello et al., 2020). This is primarily due to the higher production costs associated with traditional biosurfactant production using purified hydrophobic and hydrophilic substrates, such as purified sugars and oils, which hinder the commercialization of biosurfactants. For instance, Wang et al. (2020b) evaluated the costs and profits in the scaled-up production of sophorolipids. The estimated prices of sophorolipid crystals and syrups are USD 38,460 and USD 25,640 per tonne, respectively. However, the prices of purified glucose and oleic acids are USD 5,100 and USD 15,400 per tonne, respectively, with processing costs estimated at USD 20,000 per tonne, which already exceeds the selling price of the products. This indicates that the choice of feedstock significantly influences the profitability of SL production (Wang et al., 2020b). To further increase the potential commercial viability, feedstock should ideally be cheap and locally available throughout the year. For example, raw material costs for rhamnolipids production can account for approximately 50% of the total production cost. However, Patria et al. (2021) collaborated with the Environmental Protection Department of Hong Kong and obtained free food waste digestate as feedstock, significantly reducing the raw material cost to 10.61% of the total cost (Patria et al., 2021).

In addition to production costs, factors such as immature processes and lower yields also serve as significant constraints on the expansion of biosurfactant production (Roelants and Soetaert, 2022). Different approaches have been employed to improve biosurfactant productivity, including the utilization of cheap raw materials, optimization of culture conditions, selection of robust microorganisms, genetic modification of microorganisms, and the development of novel cost-effective downstream processes. Companies such as AmphiStar (https://amphistar.com/), V-Surf (https://vsurf.co/), and Holiferm (https://holiferm.com/) have been actively involved in optimising fermentation conditions and exploring different microbial strains to reduce production costs further (Sales da Silva et al., 2020; Maria da Gloria and Sarubbo, 2021).

## 5 Sustainable production of glycolipid biosurfactants

### 5.1 Glycolipid biosurfactants production from secondary feedstock

The increasing focus on valorization, sustainability, and green production principles has direct relevance to SDG 12 - Responsible Consumption and Production. SDG 12 aims to promote sustainable production patterns, resource efficiency, and waste generation reduction. The utilization of by-products from the industrial sector and waste streams in biosurfactant bioprocessing aligns closely with these objectives, which promotes the efficient use of resources, reduces the dependency on virgin materials, minimizes waste generation and disposal, and contributes to effective waste management and zero-waste targets (Nizami et al., 2017; Satpute et al., 2017). Moreover, to make biosurfactant production more commercially feasible, it is crucial to minimize the cost of feedstock. To realize the low-cost production of biosurfactants, the identification of suitable waste streams or industrial by-products for biosurfactant production has been extensively undertaken in the past decades.

#### 5.1.1 Glycolipids produced from hydrophilic substitutes

Glucose has been identified as the most promising feedstock for high biosurfactant production, mainly because of its role in the generation of sophorose in glycolipid molecules. Therefore, glucose-rich feedstocks, which are mostly cellulosic materials (e.g., cellulose, hemicellulose and starch), are widely used for glycolipids production. However, these feedstocks typically require pre-treatment hydrolysis to promote the release of glucose, leading to additional capital and operating costs compared to feedstocks like dairy waste and molasses. Kaur et al. (2019) studied the utilization of food waste as a feedstock for sophorolipid production by *S. bombicola*. The food waste was enzymatically hydrolyzed by glycoamylase, protease and cellulase, releasing 100 g L^-1^ of glucose and 2.4 g L^-1^ of free amino nitrogen (FAN). The food waste hydrolysate was then used in fed-batch fermentation to obtain a high titer of 115.2 g L^-1^ of sophorolipid with an overall volumetric productivity of 1.25 g L^-1^ h^-1^ (Kaur et al., 2019). Furthermore, techno-economic analysis (TEA) conducted by our research team confirmed the feasibility of this production process, with a net present value (NPV) and an internal rate of return (IRR) of US$183,598,000% and 36.17%, respectively (Wang et al., 2020b). To further reduce the biosurfactant production cost, Kumar et al. (2023) utilized a leach bed reactor (LBR) for food waste hydrolysis in rhamnolipid production. The LBR utilized a microbial community consisting of *Lactobacillus sp*., *Bifidobacter sp*., and *Proteobacteria* to provide extracellular enzymes for food waste hydrolysis. This led to the hydrolysate containing complex carbohydrates of 19.23 g L^-1^, volatile fatty acids (with alcohols) of 2.23 g L^-1^ and free amino acids of 0.083 g L^-1^ (Kumar et al., 2023). In the subsequent fermentation process, a rhamnolipid yield of 0.6—0.8 g L^-1^ was obtained (Xu et al., 2014).

Glycerol was previously reported as the preferred carbon source for rhamnolipid production. However, the utilization of agro-industrial waste as alternative feedstocks offers a more cost-effective and environmentally friendly approach (Gaur et al., 2019). Waste glycerol, a by-product of the biodiesel industry, has been increasingly utilised recently as a cheap carbon source. It typically comprises 70%–98% glycerin, accompanied by minimal amounts of fatty acids, methyl esters, fatty alcohols, and inorganic salts (Zhang et al., 2022c). Baskaran et al. (2021) used a biodiesel co-product stream (BCS) as feedstock for *P. aeruginosa* RS6 to synthesize rhamnolipid. In a 72-h shake flask experiment, they achieved a final titre of 2.73 g L^-1^ (Baskaran et al., 2021). Dobler et al. (2020) studied the influence of the C/N ratio on rhamnolipid yield using a modified *P. aeruginosa* strain -estA. Results revealed that a lower C/N ratio (50) did not provide an adequate carbon source for the microorganism, while an elevated ratio of C/N = 116 adversely affected cell growth, leading to a slower process. Ultimately, a C/N ratio of 83.2 was identified as the optimal condition. By integrating crude glycerin obtained from the soybean biodiesel industry as a carbon source, a rhamnolipid production of 17.6 g L^-1^ with a productivity of 0.0735 g L^-1^h^-1^ was achieved (Dobler et al., 2020). Moreover, there is growing interest in exploring alternative hydrophilic substitutes for glycolipid biosurfactant production, including agricultural waste and fruit waste. Table 3 provides an overview of recent studies investigating the production of glycolipid biosurfactants using various hydrophilic substitutes.

**TABLE 3:** Recent studies on glycolipid biosurfactants produced from different hydrophilic substitutes.

Microorganism	Glycolipid biosurfactant type	Hydrophilic feedstock	Hydrophobic feedstock	Nitrogen source	Fermentation conditions	Yield (g L^-1^)	Productivity (g L^-1^h^-1^)	References
*Planomicrobium okeanokoites* IITR52	Rhamnolipids	Corncob, pineapple syrup, and glycerol	—	NaNO_3_	Shake flask fermentation with 200 mL working volume, 30°C, 150 rpm, 5 days	1.5	0.0125	Gaur et al. (2022a)
*Acinetobacter calcoaceticus* BU-03	Rhamnolipids	Food waste digestate	—	—	Bioreactore fermentation with 1.2 L working volume, 55°C, 400 rpm, 1.5 vvm, 43 h	10.25	0.238	Johnravindar et al. (2022)
*Pseudomonas aeruginosa* SR17	Rhamnolipids	Jackfruit waste	—	—	Shake flask fermentation with 100 mL working volume, 37°C, 150 rpm, 120 h	2.3	0.0192	Patowary et al. (2022)
*Pseudomonas aeruginosa* RS6	Rhamnolipids	Waste glycerol from biodiesel side stream	—	NaNO_3_	Shake flask fermentation with 200 mL working volume, 35°C, 180 rpm, 72 h	2.73	0.0379	Baskaran et al. (2021)
*Pseudomonas aeruginosa-*estA	Rhamnolipids	Crude glycerin	—	NaNO_3_	NA	17.6	0.0735	Dobler et al. (2020)
*Starmerella bombicola* ATCC 22214	Sophorolipids	Food waste	Oleic acid	Yeast extract	Bioreactore fermentation with 1.5 L working volume, 30°C, 600 rpm, 2.67 vvm, 96 h	137.5	1.432	To et al. (2023)
*Starmerella bombicola* ATCC 22214	Sophorolipids	Corn straw	Oleic acid	HH_4_NO_3_	Shake flask fermentation, 30°C, 160 rpm, 7 days	27.45	0.163	Yu et al. (2021)
*Rhodotorula babjevae* YS3	Sophorolipids	Corn distillers dried grains with solubles	—	—	Shake flask fermentation, 19°C, 200 rpm, 72 h	19.27	0.268	Sen et al. (2021)
*Rhodotorula babjevae* YS3	Sophorolipids	Rice distillers dried grains with solubles	—	—	Shake flask fermentation, 19°C, 200 rpm, 72 h	17.81	0.247	Sen et al. (2021)
*Starmerella bombicola* ATCC 22214	Sophorolipids	Food waste hydrolysate and glucose	Oleic acid	—	Fed-batch fermentation with *in situ* separation, 1.5 L working volume, 30°C, 600 rpm, 2.67 vvm, 240 h	—	2.43	Wang et al. (2020a)

#### 5.1.2 Glycolipids produced from hydrophobic substitutes

The utilization of hydrophobic secondary materials as carbon sources for glycolipid production has gained attention in recent studies, and waste oil from various sources become a potential substitute. Perez-Armendariz et al. (2019) investigated the potential of waste canola oil as a low-cost and environmentally friendly substrate for the production of rhamnolipids by *P. aeruginosa*. By conducting a 2^3^ full factorial design experiment, they identified nitrogen source was a crucial factor, as the use of NaNO_3_ rather than (NH_4_)_2_SO_4_ led to a 30-fold increase in production yield (Perez-Armendariz et al., 2019). In another study conducted by Pathania and Jana (2020), the co-utilization of glucose and waste frying oil as substrates exhibited significant advantages compared to using single carbon sources. This co-substrate utilization not only positively influenced cell growth but also impacted quorum sensing and modulated the biosynthetic pathway, leading to an improvement in rhamnolipid production. However, they also found that a glucose concentration above 3% w v^−1^ in the mixed substrate was ineffective for rhamnolipid production. After the condition optimization by response surface methodology, a rhamnolipids production of 6.3 g L^-1^ was achieved, and higher substrate diversion towards the product than towards the cell growth was observed (Pathania and Jana, 2020). Similarly, other hydrophobic feedstocks such as waste cooking oil and sunflower oil cake have been used in the fermentation production of various biosurfactants, including sophorolipid (Jadhav et al., 2019; To et al., 2022), rhamnolipid (Li, 2017; Bakur et al., 2019) and mannosylerythritol lipid (Bakur et al., 2019; Niu et al., 2019). Table 4 shows some recent studies on glycolipid biosurfactants produced from different hydrophobic substitutes.

**TABLE 4:** Recent studies on glycolipid biosurfactants produced from different hydrophobic substitutes.

Microorganism	Glycolipid biosurfactant type	Hydrophilic feedstock	Hydrophobic feedstock	Nitrogen source	Fermentation conditions	Yield (g L^-1^)	Productivity (g L^-1^h^-1^)	References
*Pseudomonas aeruginosa* FA1	Rhamnolipids	—	Peanut meal	NaNO_3_	Solid-state fermentation, 35°C, 6 days	3.469	0.0241	Zhao et al. (2023)
*Pseudomonas aeruginosa* M4	Rhamnolipids	—	Waste cooking oil	Tryptone	Shake flask fermentation with 30 mL working volume, 35°C, 180 rpm, 132 h	1.120	0.00848	Shi et al. (2021)
*Pseudomonas aeruginosa* NJ2	Rhamnolipids	Glucose	Waste frying oil	HH_4_NO_3_	Shake flask fermentation with 100 mL working volume, 30°C, 150 rpm, 96 h	6.3	0.0656	Pathania and Jana (2020)
*Pseudomonas aeruginosa*	Rhamnolipids	—	Waste canola oil	NaNO_3_	Shake flask fermentation with 100 mL working volume, 37°C, 200 rpm, 14 days	3.585	0.0107	Perez-Armendáriz et al. (2019)
*Pseudomonas aeruginosa* MTCC7815	Rhamnolipids	—	Waste cooking oil	NaNO_3_	Shake flask fermentation with 100 mL working volume, 25°C, 5 days	11	0.0917	Sharma et al. (2019)
*Starmerella bombicola* Y-6419	Sophorolipids	Glucose	Household residual cooking oil	Rice bran hydrolysate	Shake flask fermentation, 30°C, 200 rpm, 216 h	51	0.236	Rocha et al. (2023)
*Starmerella bombicola* ATCC 22214	Sophorolipids	Glucose	Bakery waste oil	Yeast extract	Batch bioreactor fermentation with 1.5 L working volume, 30°C, 600 rpm, 2.67 vvm, 144 h	96.4	0.446	To et al. (2022)
*Starmerella bombicola* ATCC 22214	Sophorolipids	Potato scraps	Rapeseed oil	Potato scraps	Fed-batch bioreactor fermentation with 1 L working volume, 30°C, 200–800 rpm, 4–5 mL/min air flow, 5 days	77.8	0.684	Wongsirichot et al. (2022)
*Starmerella bombicola* ATCC 22214	Sophorolipids	Glucose	Waste cooking oil	Yeast extract	Fed-batch bioreactor fermentation with 2 L working volume, 25°C, 400 rpm, 1 vvm, 288 h	315.6	1.096	Kim et al. (2021)
*P. aphidis* ZJUDM34	Mannosylerythritol lipid	—	Waste cooking oil	NaNO_3_, yeast extract	Shake flask fermentation, 28°C, 180 rpm, 10 days	55.00	0.229	Niu et al. (2019)

#### 5.1.3 Glycolipids produced from other secondary materials

In addition to the pursuit of cost-effective carbon sources, scholars are also actively investigating the utilization of affordable nitrogen sources and exploring novel technologies in sustainable glycolipids production. Corn steep liquor (CSL), a by-product of the corn wet-milling industry, is widely recognized as a valuable nitrogen source in biotechnological processes. Apart from amino acids and proteins, CSL is enriched with vitamins, minerals, and varying amounts of carbohydrates, making it a low-cost carbon source as well. Olive mill wastewater (OMW), on the other hand, has garnered attention as an inexpensive source of long-chain fatty acids for rhamnolipid production. By combining these two secondary feedstocks together, Correia et al. (2022) observed a high rhamnolipid production of 269 mg L^-1^ in bioreactor fermentation. However, the rhamnolipids produced in the CSL + OMW medium showed weak emulsifying activity, which can be explained by the different relative abundance between mono-rhamnolipid and di-rhamnolipid in the product (Correia et al., 2022).

In order to simplify the separation of rhamnolipids from the fermentation broth as well as improve the mass transfer rate in the aqueous-organic mixture, Liu et al. (2023) proposed a combined strategy of cell immobilization and oil emulsion. By immobilizing bacterial cells into alginate-chitosan-alginate microcapsules, they effectively enhanced biomass tolerance to environmental disturbances and facilitated biomass recovery in continuous processes. Furthermore, they significantly improved bacterial utilization of oily carbon sources in the fermentation broth by the emulsification effect of the biosurfactant. Through the integration of fermentation and foam fractionation, they further enhanced the fractionation efficiency of rhamnolipids while reducing the adverse toxicity of foam on cell metabolism. Ultimately, by combining all these techniques, they achieved a high yield of 7.18 g L^-1^ using 20% fried oil as the substrate in a fermentation-foam fractionation coupling system (Liu et al., 2023). Similarly, other novel technologies have been explored for different biosurfactant production, such as the utilization of wheat straw as support in solid-state fermentation of sophorolipids production (Rodríguez et al., 2021).

In conclusion, the exploration of glycolipid biosurfactant production from secondary feedstocks has opened up new avenues, which contribute to the development of more sustainable and economically viable glycolipid biosurfactant production methods. Although research and studies have demonstrated promising results in terms of glycolipid yields and properties, further investigations are needed to optimize the production process and enhance productivity for industrial-scale production.

### 5.2 Challenge of adopting secondary materials for sustainable biosurfactant production

#### 5.2.1 Challenges of process adaptability and optimization

The secondary feedstock may contain some ingredients that inhibit the fermentation process, resulting in limited biomass and low production yield. Researchers have employed various strategies to mitigate the inhibitory effects and optimize biosurfactant production from these feedstocks. For example, Konishi et al. (2015) used sulphuric acid to hydrolyse crushed corncobs and produced sterilized hydrolysate by autoclaving. The authors found that extending the autoclave duration and increasing the sulphuric acid concentration resulted in a dark brown hydrolysate, which was linked to an increase in the Maillard reaction and furfural concentration. To minimize furfural release, the sulphuric acid concentration was optimized to 1% w v^−1^. This resulted in an improved sophorolipid production, with a titre of 50.5 g L^-1^ and volumetric productivity of 0.421 g L^-1^ h^-1^ after 120 h of cultivation in a batch bioreactor. Further optimisation involved the addition of ammonium nitrate, which reduced the inhibitory effect of autoclaving and further improved the sophorolipid titre to 49.2 g L^-1^ in 96 h of cultivation, leading to higher volumetric productivity of 0.513 g L^-1^ h^-1^ (Konishi et al., 2015). In another study by Yu et al. (2021), solid acid treatment, autoclaving, and enzymatic hydrolysis were employed to produce hydrolysate from corn stoves. Activated carbon and vacuum evaporation were used to remove the furfural, pigment, and volatile inhibitors from the hydrolysate, generating a concentrated hydrolysate suitable for sophorolipid production. These strategies increased the final sophorolipid titre from 17.17 g L^-1^–27.54 g L^-1^, corresponding to productivity of 0.163 g L^-1^ h^-1^ in 7 days of shaking flask cultivation (Yu et al., 2021). To et al. (2023) also identified lactic acid as the major inhibitor in the food waste hydrolysate in sophorolipid bioprocessing. Since lactic acid is water soluble, an additional water-washing step was used to remove the inhibitor and restore the fermentation (To et al., 2023). Similarly, the presence of furfural, acetate, and formate in the lignocellulosic biomass was identified as inhibitors that can impede MEL fermentation by *Moesziomyces antarcticus*. However, the addition of D-xylose was found to improve MEL production to a level comparable to the control without inhibitors (Santos et al., 2019).

The industrial-scale production of biosurfactants is attracting significant interest from various stakeholders (Dierickx et al., 2022). Very recently, a European Horizon 2020 research and development project titled ‘Biosurfactants Production from Industrial Food Waste Feedstocks as Novel Functional Ingredients for Consumer Products’ (acronym: WASTE2FUNC). This project starts from 2021 which is coordinated by Bio Base Europe Pilot Plant and Department of Biotechnology, Faculty of Bioscience Engineering, Ghent University, Belgium, with the collaboration of several research institutes, academic, and industrial partners in Europe, Israel and Hong Kong. The aim of this project is to demonstrate the production of biosurfactants and lactic acid from food waste from agriculture, the food industry, supermarkets and restaurants. The corresponding project, which is currently conducted by School of Energy and Environment at City University of Hong Kong, titled ‘Development of One-step Food Waste Biorefinery via Novel Bioreactor Design, Functional Stain Adaptive Laboratory Evolution and Genetic Engineering’, began in December 2021. The project aims to develop a food waste–derived biosurfactant via a novel fermenter design and the genetic engineering of a robust yeast to explore the bioeconomic viability of waste biorefineries. The proposed work signifies a new direction in the field of heterologous enzyme expression in an unconventional yeast strain and integrated hydrolysis–fermentation bioprocesses. In addition, environmental and economic effects/benefits associated with this new sustainable waste-based biorefinery production methodology will be evaluated.

#### 5.2.2 Challenges of socio-economic impacts and sustainability benefits

**FIGURE 5:**
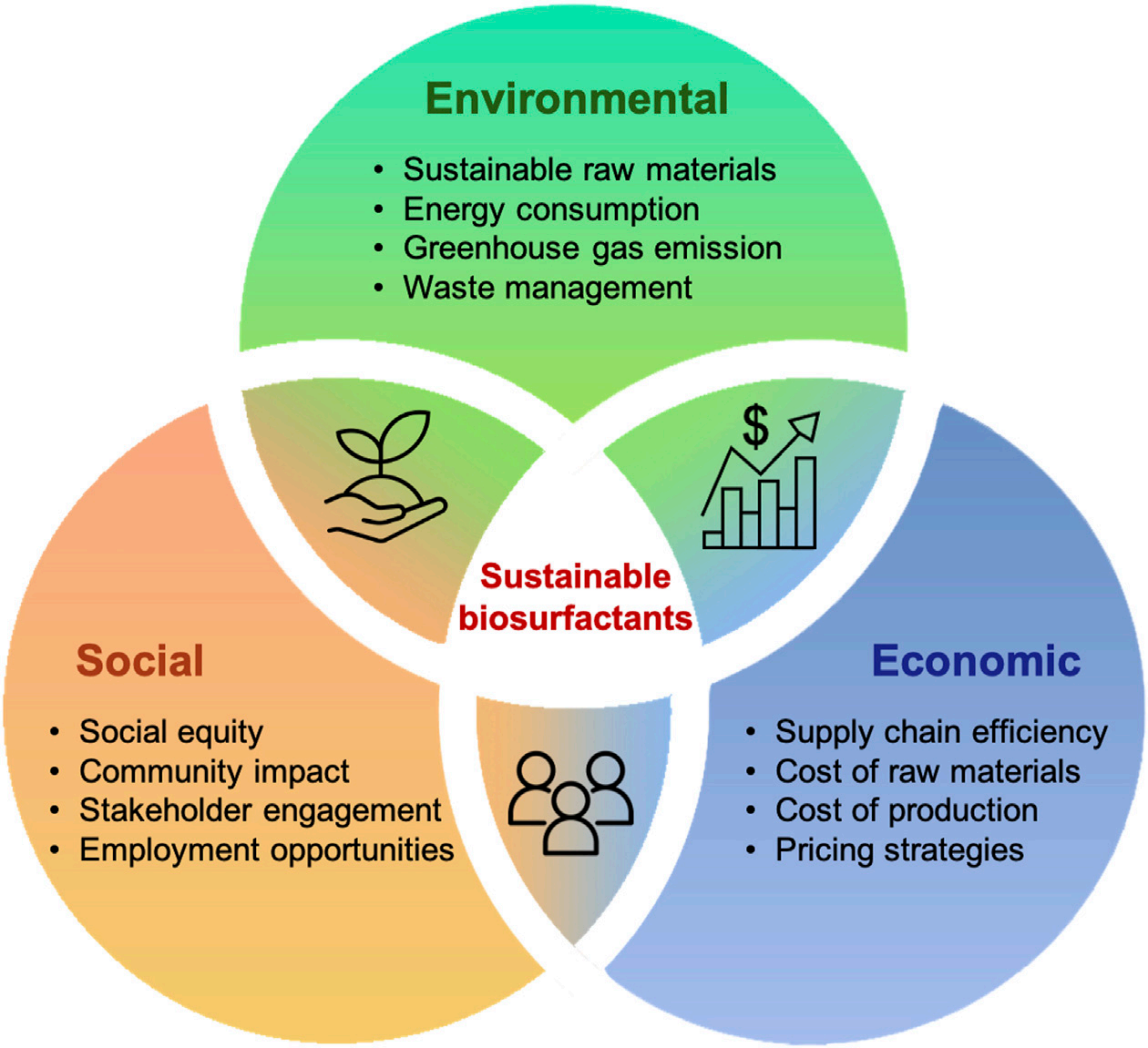
Challenges in the production of sustainable biosurfactants: environmental, social, and economic perspectives.

The development of biorefineries is seen as a pivotal strategy, offering a win-win scenario, as most chemicals can be synthesized using specific building blocks that can be substituted with their bio-based equivalents (Kannegiesser, 2008). This shift not only highlights the economic feasibility of replacing fossil fuels but also contributes to the reduction of greenhouse gas (GHG) emissions (Davis et al., 2013). Although the transformation of biomass into bio-products through biorefineries brings substantial advantages, this emerging technology also faces challenges across the three pillars of environmental benefits, social impact and economic viability (Figure 5). The integrated performance of the biorefinery plays a crucial role, necessitating a comprehensive analysis to evaluate its impact on these pillars (PrasaraA and Gheewala, 2017). In this regard, life cycle assessment (LCA) and techno-economic analysis (TEA) emerge as the most commonly used methodologies to conduct a thorough and holistic assessment of biorefinery operations.

##### 5.2.2.1 Life cycle assessment (LCA) of biosurfactants

LCA methodologies are widely recognized techniques for evaluating the inputs, outputs, and potential environmental consequences of a product system throughout its entire life cycle (Sala et al., 2021). By conducting an LCA, it becomes possible to obtain a comprehensive understanding of the environmental performance of the product system, which provides valuable insights for establishing guiding principles to achieve sustainable consumption and production (Valdivia et al., 2021).

A dynamic life cycle assessment (*dLCA*) was used to iteratively evaluate the environmental impact of SL production as technology evolved (Chopra et al., 2019). Three traversals were conducted focusing on different procedures of the production process. Results indicated that food waste was the most suitable feedstock source for SL production, outperforming textile and bakery waste (Hu et al., 2021b). Utilizing fed-batch fermentation of food waste integrated with *in situ* separation techniques resulted in lower environmental impacts, mainly due to the relatively low cumulative energy demand (CED) and global warming potential (GWP) (Hu et al., 2021a). In addition, energy consumption was identified as a critical hotspot in SL production, accounting for over 80% of the total impact. Biorefineries should prioritize energy-saving measures to reduce their carbon footprint and foster sustainability (Balina et al., 2023). As for the downstream stages, increasing the packaging volume was an effective choice for reducing the environmental impacts of biosurfactant production, resulting in a decrease of 8%–38% in total impacts with a 25% reduction in global warming potential (de Lapuente et al., 2022). Apart from optimizing process workflows, LCA is also employed to compare the environmental impacts of various products. Schonhoff et al. (2022) revealed that when using substrates from the sugar industry, mannosylerythritol lipids production exhibited lower environmental impacts than rhamnolipids due to their advantageous microbial properties and process designs. This finding highlights the potential advantages of MEL as a more sustainable option in terms of environmental performance compared to rhamnolipids (Schonhoff et al., 2022).

However, Longati et al. (2023) maintain a conservative stance towards biosurfactants because they present a higher GWP than surfactants, presenting emissions from 7.3 to 17.1 kg CO_2_ eq./kg, while surfactants have emissions between 0.9–6.1 kg CO_2_ eq./kg (Longati et al., 2023). Although the substantial disparities in conclusions primarily stem from divergent data sources, the large-scale production of biosurfactants, as an emerging technology, still faces challenges and bottlenecks that need to be overcome.

##### 5.2.2.2 Techno-economic analysis (TEA) of biosurfactants

LCA is always complemented by TEA, a powerful method to conduct the economic analysis of a production system by modelling the plant process at a high level of detail (Campion et al., 2023). The integration of these two methods is commonly referred to as life cycle sustainability assessment (LCSA), which considers all three pillars throughout the life cycle of the process, guiding production towards sustainability (Ciroth et al., 2011).

A study conducted by our research team demonstrated the sustainable sophorolipids production via biorefinery utilizing food waste in Hong Kong (Wang et al., 2020b). The study revealed that 100 MT of food waste can result in the production of either 93 MT of SL crystals or 120 MT of SL syrup. Comparing various bioproducts, producing SL crystals from food waste proved to be more profitable than producing succinic acid or lactic acid, primarily due to the higher commodity price of SL. Additionally, SL production was deemed more sustainable, as the purification of lactic acid and succinic acid required more complex downstream processes, consuming greater energy and chemicals. When producing SL via solid-state fermentation (SSF) using winterization oil cake (WOC) and molasses as secondary feedstocks, the physical properties of substrates and supports, such as bulk density and water-holding capacity, play a crucial role in determining process costs beyond the expenses incurred for substrate purchase and process yields (Martinez et al., 2022). In production processes, electricity costs constitute a significant proportion of variable costs, reaching as high as 78.22% (Moutinho et al., 2021). However, simulation of different process scenarios showed that selecting aeration units with high oxygen transfer rates and adjusting power input to match oxygen uptake can significantly decrease electricity consumption (Oraby et al., 2022). The socio-economic advantages of sustainable biosurfactants are also proved in another study by Schonhoff et al. (2023), which showed that the use of molasses as a substrate leads to lower financial expenditures (35%–55%). Furthermore, specific supply chains, particularly those involved in the manufacturing of chemical products like solvents, were identified as the primary drivers of social impacts (Schonhoff et al., 2023).

#### 5.2.3 Challenges of data acquisition and regulatory barriers

LCSA offers several advantages for decision-makers and stakeholders; however, it faces challenges in providing detailed information to perform life cycle costing (LCC), environmental life cycle assessment (E-LCA), and social life cycle assessment (S-LCA). Although different software companies have provided databases, the available information is limited because the industrial-scale processes are absent, which translates to insufficient data for accurate assessments (Dragone et al., 2020; Mariana et al., 2021). Ensuring the economic viability of biorefineries is essential to guarantee their success and attract investment, but the cost estimation for engineering, procurement, and construction aspects also poses challenges that can affect the quality of analysis (Zeug et al., 2021). It should be noteworthy that even with proper methodologies in place to assess sustainability across the three pillars, the lack of data remains a limitation in conducting comprehensive evaluations. Furthermore, regulatory barriers also impede their market penetration, posing difficulties when compared to their well-established fossil-based counterparts (Leibensperger et al., 2021).

In summary, the establishment of a robust bioeconomy framework relies on the integration of biorefinery, sustainable production, and biomass utilization. Nevertheless, consolidation of technologies and information for socio-economic impacts and sustainability assessment, as well as supply chain issues, must be overcome in order to link the biorefinery to the productive stage. Therefore, more effort and government policies must be introduced to achieve the SDGs targets agreed in the 2030 agenda.

## 6 Conclusion

Biosurfactants have recently garnered augmented attention due to their remarkable attributes: high biodegradability, low toxicity, and resilience to extreme pH and temperature conditions, surpassing their fossil-derived counterparts. Among these versatile biomolecules, glycolipids, which are classified as low-molecular-weight biosurfactants, excel in diminishing surface and interfacial tension. Notably, rhamnolipids, trehalose lipids, sophorolipids, and mannosylerythritol lipids stand out as the most prominent representatives. Despite the broad spectrum of applications for glycolipids, including bioremediation, food processing, petroleum refining, biomedical applications, and agriculture, their production cost remains the principal impediment in outperforming synthetic surfactants. Although the integration of secondary feedstocks presents a potential avenue for enhancing the sustainability of glycolipid production, the utilization of such feedstocks in industrial settings remains limited due to the heterogeneous composition of these feedstocks. Addressing this issue requires substantial efforts to refine process flow and enhance productivity. It is believed that with proper optimization of the use of secondary feedstocks in biosurfactant production, the balance between environmental benefits, economy and society can be achieved. With these advancements, the production of glycolipids can become more sustainable, contributing to a greener and more environmentally friendly surfactant industry.
